# *Balony:* a software package for analysis of data generated by synthetic genetic array experiments

**DOI:** 10.1186/1471-2105-14-354

**Published:** 2013-12-04

**Authors:** Barry P Young, Christopher JR Loewen

**Affiliations:** 1 Department of Cellular and Physiological Sciences, Life Sciences Institute, University of British Columbia, 2350 Health Sciences Mall, Vancouver, British Columbia V6T 1Z3, Canada

**Keywords:** Yeast, Array, Image analysis, Software

## Abstract

**Background:**

Synthetic Genetic Array (SGA) analysis is a procedure which has been developed to allow the systematic examination of large numbers of double mutants in the yeast *Saccharomyces cerevisiae.* The aim of these experiments is to identify genetic interactions between pairs of genes. These experiments generate a number of images of ordered arrays of yeast colonies which must be analyzed in order to quantify the extent of the genetic interactions. We have designed software that is able to analyze virtually any image of regularly arrayed colonies and allows the user significant flexibility over the analysis procedure.

**Results:**

“*Balony*” is freely available software which enables the extraction of quantitative data from array-based genetic screens. The program follows a multi-step process, beginning with the optional preparation of plate images from single or composite images. Next, the colonies are identified on a plate and the pixel area of each is measured. This is followed by a scoring module which normalizes data and pairs control and experimental data files. The final step is analysis of the scored data, where the strength and reproducibility of genetic interactions can be visualized and cross-referenced with information on each gene to provide biological insights into the results of the screen.

**Conclusions:**

Analysis of SGA screens with *Balony* can be either automated or highly interactive, enabling the user to customize the process to their specific needs. Quantitative data can be extracted at each stage for external analysis if required. Beyond SGA, this software can be used for analyzing many types of plate-based high-throughput screens.

## Background

The development of high-throughput array-based technologies such as SGA analysis [1,2] has led to a rapid increase in the popularity of systematic genome-wide genetic interaction screening in yeast. In a typical SGA experiment, a query yeast strain containing a single specified gene deletion is mated to the yeast haploid deletion collection of ~4800 individual gene deletion mutants arrayed in colonies on agar plates. Following diploid selection, sporulation and selection of haploids, a complete set of double mutant strains is generated, which can be used to define the spectrum of genetic interactions for the query gene, thus providing unbiased information about its function in the cell. In recent years, the availability of relatively low-cost robotic platforms such as the Singer RoToR HDA (http://www.singerinstruments.com) has led to the uptake of this technology by an increasing number of non-specialist laboratories. However, the lack of availability of specialized software for the analysis and quantitation of array colonies has hampered these efforts.

In an SGA experiment, after completion of the robotic pinning steps the experimenter is presented with a substantial number of agar plates containing ordered arrays of differently sized single and double mutant yeast colonies. The relative size of the colonies represents the fitness of each strain, which can be used as measure of the strength of a genetic interaction. In order derive meaningful genetic interaction data from these arrays, the size of each colony needs to be precisely measured and the data normalized and compared with an appropriate control. Given that a single SGA experiment can result in numerous (often up to 50) replicates of arrays, each containing up to 1,536 colonies per array, it is desirable that such analyses can be carried out in a high-throughput manner with as much automation as possible. Nevertheless, most experimenters will want some degree of control over the measurement process, so providing a level of interactivity will improve overall confidence in the final results.

One problem we encountered when we first attempted to analyze images using existing software packages (e.g. “ScreenMill” [3]) was that they tended to be designed with a particular image format in mind. Although they were effective at analyzing sample images provided with each program, we were unable to analyze images that we had obtained ourselves. Furthermore, if the software was unable to identify the colonies on a plate, the program would fail with little recourse available. While these programs present a simple interface to the user, it is not possible to adjust the imaging parameters that might enable successful analysis of an image.

To that end, we sought to develop a program “*Balony*”, that would be able to analyze images regardless of their specific properties, and with the flexibility to utilize arrays of any possible format. Although we find that the default settings used by *Balony* are suitable for analyzing most plates, the ability to manually adjust image analysis parameters allows users to quantify even the most troublesome images.

As a demonstration of the flexibility of our image analysis engine, we were able to use *Balony* to successfully quantify the example image plates provided with both ScreenMill [3] and SGAtools [4], while neither of these packages were able to analyze the sample images provided by any of the other programs.

We also sought to design a program that would enable the complete analysis of a screen, from scanned images of plates to an interactive display of genes of interest, all from a single interface. While both ScreenMill and SGAtools necessarily involve using external web services to carry out some or all portions of their data analysis, *Balony* operates as a single, stand-alone window making it easy to switch between modules to monitor the effects of adjusting settings. Although this software is primarily aimed at analyzing high-throughput experiments in yeast, it could also be employed for use with any system that utilizes high-density arrays of microbial colonies.

## Implementation

**Figure 1:**
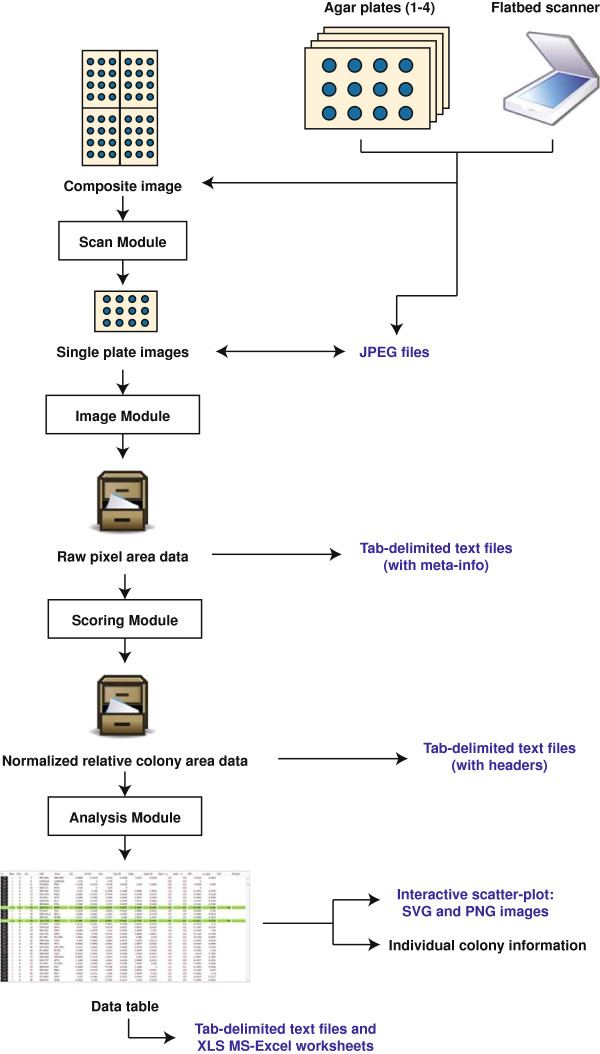
**Data-flow through *Balony*.** Images are acquired using a flatbed scanner, either singly or in composites of up to four plates; in the latter case these images are segmented into single images. The imaging module measures raw pixel areas and saves this information as text files (one file per plate). These files are collated by the scoring module and normalized; the data saved as tab-delimited text files; either one per set or as an averaged file. The analysis module facilitates interrogation of the screen data. In addition to saving the quantitative data in formats for use in spreadsheet applications, images of the plot of colony area ratios can be saved in bitmap and structured variants.

*Balony* is a stand-alone Java program, which uses libraries from various sources, most notably the ImageJ library for image manipulation [5], and The Apache Commons Mathematics Libraries for statistical analysis. The program has a modular structure, shown in Figure 1. Data files are generated at each stage of the analysis and can be inspected at will. If a user so chooses, they can merely use parts of the *Balony* package to measure colony sizes and perhaps perform normalization, and then use their own scripts or programs to further score their data.

**Figure 2:**
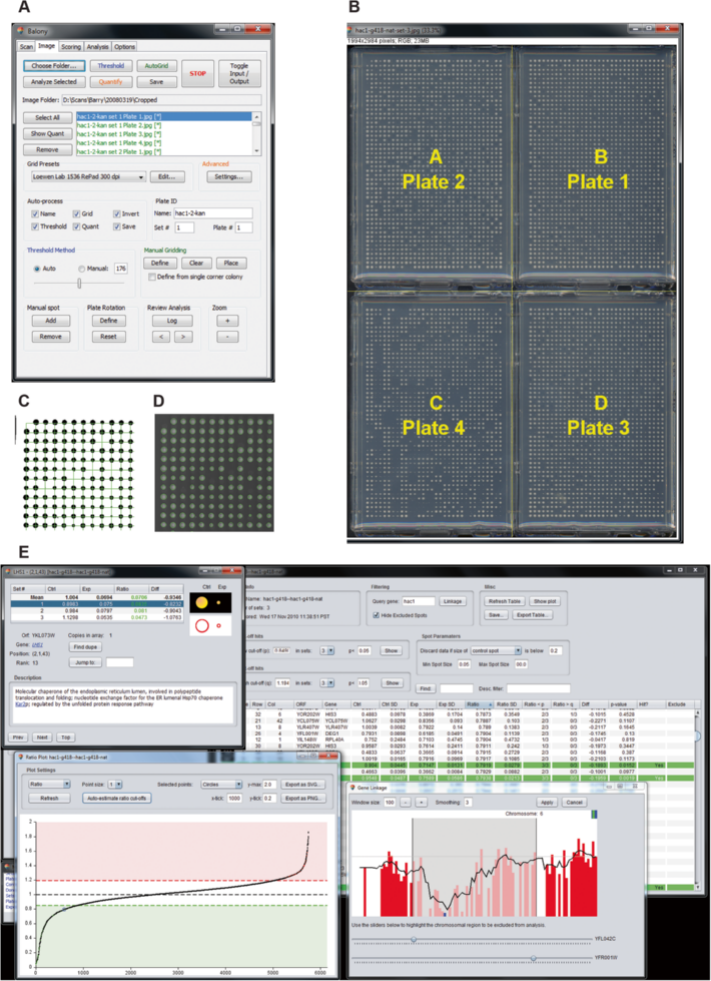
**The balony program. A**. Screenshot of the graphical user interface, in this case showing the Imaging module. **B**. A composite image of four plates demonstrating how it would be divided into separate images. **C**. A portion of an inverted, thresholded image overlayed with the array grid in green. **D**. A portion of the same image showing successfully quantified colonies. **E**. Analysis module.

The data flow starts with composite images of multiple plates or single images of individual plates. In the case of composite images, the “Scan” module converts these to images of single plates. This can speed up the image acquisition stage of the analysis by allowing the user to capture images of up to four plates at a time. These images are then analyzed by the “Image” module which produces a text file containing raw data listing the pixel area of each colony in each image. These text files are then used as inputs for the “Scoring” module which pairs control and experimental data sets and performs normalization to produce one or more tab-delimited text files containing the normalized colony sizes for the experiment. This data can then be viewed directly (e.g. by loading into a spreadsheet) or analyzed using the “Analysis” module. This enables collation of multiple sets of data and further refinement, e.g. by removal of genes linked to the query gene in an SGA experiment. Cut-off values to determine “hits”, p-value thresholds and reproducibility across data sets can also be defined to precisely determine “hit lists” of genes. The Analysis module enables the direct inspection of individual data points, providing gene information from the *Saccharomyces* Genome Database (SGD) [6]. The main window of the program is divided into five tabs which are used to sequentially analyze data (Figure 2A).

### Image segmentation: the scan tab

The “Scan” section of *Balony* allows users to take composite images of multiple plates and subdivide them into separate images for analysis (Figure 2B). We find that images of plates are best captured using a flatbed scanner as the reduced depth of field of a scanner compared to a digital camera results in less optical distortion of the images. It is advisable to scan plates with a black background (e.g. card or cloth) to improve contrast between the colonies and the agar.

We find that a final resolution of 300 dots per inch (dpi) is sufficient for most applications, although for ultra-high density experiments using arrays with 6144 colonies per plate (cpp), higher resolutions may be required. In general, processing time increases with image resolution, and the extra information above 300 dpi is unlikely to provide more robust data as the inherent variance in the size of yeast colonies will be more significant than any additional fine detail gained.

When performing SGA experiments we use a variety of terms to describe the components of an experiment. Each array consists of a number of agar *plates*. For example, the haploid yeast Deletion Mutant Array (DMA) consists of four plates when arrayed at a density of 1536 cpp, which we would simply refer to as plates 1-4. Replicates of each plate are termed *sets*. Thus in a typical SGA experiment, one might produce three replicates of a query strain crossed with the DMA, i.e. 3 sets of 4 plates.

To ease downstream processing of images it is important that files are named systematically (Table 1). Input files should be named according to the template “screen_setx.jpg”, which will result in output files with the format “screen_set-x_plate-y.jpg”. If there are more than four plates per set, an offset value can be included in the file name which will be added to each plate number. For example, a file named “yfg1_set1_[4].jpg” will by default number plates starting at 5 (i.e. 4 + 1). However, plates can also be scanned individually, in which case each set of plates should be named in the format “screen_setx_platey.jpg”.

**Table 1:** File name handling in balony

**Input file name**	**Output file names**
Yfg1_G418_set-1.jpg	Ygf1_G418_set-1_plate-1.jpg
	Ygf1_G418_set-1_plate-2.jpg
	Ygf1_G418_set-1_plate-3.jpg
	Ygf1_G418_set-1_plate-4.jpg
Yfg2_URA_set-3_[4].jpg	Yfg2_URA_set-3_plate-5.jpg
	Yfg2_URA_set-3_plate-6.jpg
	Yfg2_URA_set-3_plate-7.jpg
	Yfg2_URA_set-3_plate-8.jpg

Plates can be rotated automatically to ensure the correct orientation with respect to gene index files as the subsequent analysis steps assume that the top left position of a plate is identified as “row 1, column 1”. Images can also be resized and, if they are not already, converted to greyscale. By default, the individual plate image files are named according to a scheme to enable tracking of the plate and set number, which is recommended to ensure easy identification of plates in the Colony Measurement section (see below). However, the “base name” and each individual plate name can be overridden if necessary.

An entire folder of composite images can be analyzed in batch mode to reduce the amount of user input required at this stage. Processing time is dependent on a number of factors, such as the resolution of the input images and the speed of the computer, but should not take more than a few minutes for a typical screen.

The user can define different mappings which define how the position of a plate in the scanned image is converted into a plate number based on an array index. The default setting is shown in Figure 2B and is ideally suited to the deletion collection maintained at 1536 cpp format as this consists of exactly four plates. It is important to remember that a scanned image will usually be reflected about its *y*-axis, so it is advisable that the first time a user uses this module that they visually check that the plates are correctly assigned and oriented in order to prevent mishaps further downstream. If the mapping needs to be changed from the default setting, the new assignments will be remembered for future analyses.

### Colony measurement: the image tab

*Balony* uses a multi-step process to measure colony sizes on individual plate images. Each step can be customized with varying parameters which enables a high degree of compatibility with plates from a variety of sources. The measurement process identifies colonies as elliptical objects, measures the pixel area of each object, and assigns the object to a grid position. The raw data (grid row, grid column and colony area) are saved for subsequent normalization, scoring and analysis. This process can be automated completely, requiring little to no user input, but if this approach is not proving fruitful, each plate can be analyzed manually using a variety of tools to give fine-grained control of the analysis process.

This panel shows a list of image files in the currently selected folder. A colour-coding and suffixing scheme is used to help the user identify which files have been analyzed. If a file has not yet been analyzed, then the filename will simply be displayed in black. If a file has been successfully analyzed, then it will be displayed in green and suffixed with [*]. If a file has been analyzed, but with a certain number of colonies that could not be correctly measured (“bad spots”) then it will be displayed in orange and suffixed with [?], as long as the number of bad spots is below a threshold value. This indicates that a plate has a small number of imperfections that may warrant closer inspection. If the number of bad spots is above the threshold value the file name will be displayed in red and suffixed with [!]. This is usually indicative of a plate which had significant defects, or the program was unable to analyze automatically. The steps necessary to analyze an image are described below.

#### Format correction

The image measurement process requires yeast colonies as black ellipses on a white background. Upon opening, images will be converted automatically to greyscale if they are not already in this colour format. As the colonies are usually lighter than the background, the image then needs to be inverted. This can be carried out automatically for each plate and is recommended.

Upon loading an image, *Balony* will attempt to decipher the file name to determine a name for that particular experimental set as well as the corresponding set and plate number. This unique name will be the same for all plates across sets for an individual experiment and is generated by stripping away the “set” and “plate” parts of the image file name. It is important that this information is consistent between all plates from a given experiment as the information is written into the meta-data saved after each image is analyzed and is used by the scoring module to identify plates of the same experiment. However, this option can be disabled if so required.

#### Thresholding

Images are converted to black-and-white using a procedure known as “thresholding”, which separates the agar plate background from the yeast colonies. In this process each pixel in the image is converted to either black or white depending on whether it falls below or above a defined grey level. The images at this stage are stored at a colour depth of eight bits per pixel, so the grey level will have a value of between 0 and 255. As in other programs, *Balony* can automatically define this threshold level using an algorithm based on a digital histogram of the image. Additionally, we have provided an option for the user to manually specify this grey level which can salvage the analysis of an image which would otherwise fail if automatic thresholding is not successful. Generally, if the thresholding is not successful, it is because the grey level selected is too high, resulting in the plate background merging with the yeast colonies. There is an option to automatically attempt to re-analyze the image with decreasing threshold values until the plate is successfully analyzed. However, care must be taken with this as if too low a value is used then there is a danger of discarding colony size information.

#### Gridding

Arrays of yeast are indexed by identifying each strain in terms of its row and column position in a grid, and optionally, a plate number. Therefore, a gridding step is required to identify the region of the image that contains the arrayed colonies. *Balony* contains extensive controls not found in other programs to assist in the correct placement of the grid, so that even if an array contains unusual features that may make automatic grid identification difficult (such as blank rows or columns, or very small colonies) manual intervention can resolve this.

The software is supplied with a number of grid presets corresponding to the most commonly used formats in use, namely 96, 384 and 1536 cpp. New presets can be defined and calibrated from sample plates. *Balony* can usually automatically determine the grid position using a particle identifying routine; however, if a plate is proving problematic, the user can manually specify the position of the grid.

The first time a user runs the program and loads an image, they will be prompted to either use an existing preset, or define a new one that matches their particular image acquisition platform. The latter option is recommended as it allows for a more precise fit for different types of imaging hardware. The user will be prompted to enter the dimensions of the array (i.e. the number of rows and columns), and then draw a box on the plate image to indicate the boundaries of the array. Following this they will be prompted to name and save the settings derived from this. The most important values from this are the mean spacing (in pixels) between colonies in the horizontal (*dx*) and vertical (*dy*) dimensions.

Should a user not wish to use the automatic gridding process, or find it ineffective for their plates, they can indicate the position of the array manually, either by dragging a box from one corner to an opposite diagonal corner; or by positioning a grid of fixed dimensions.

After the gridding process has been completed, a grid is drawn over the thresholded image in green (Figure 2C). If required, this grid can be moved into a different position using the cursor keys. The user can decide if the gridding process should proceed automatically after a plate has been thresholded. This generally speeds up the quantification process, but it may be advisable not to use it for the first few images so that the user has an opportunity to observe the different steps involved.

#### Colony assignment and measurement

Next, colony sizes are measured by analyzing all particles within the grid array and mapping them to their nearest (row, column) position within the array. Parameters can be set to ensure certain criteria are met for a particle to be identified as a colony, including minimum pixel size, circularity and deviation from the grid position. If a grid position appears not to have a colony present in that position, the program will re-scan that position to look for the presence of an object. This is sometimes necessary because overgrown colonies can and merge with neighbouring colonies and no longer appear as a discrete entity.

This process is usually sufficient to identify the colonies on a plate. However, if there are many grid positions that appear to contain something that does not satisfy the minimum criteria for a yeast colony, the software will offer the option of a low-stringency pass that will attempt to quantify the amount of growth occurring in a position, regardless of its circularity. Care should be taken with this option as blemishes on the plate surface may then be counted as colonies.

When this process is complete, the successfully quantified colonies are shown by outlining each colony in green over the original input image (Figure 2D). The user can toggle between this final image and the gridded, thresholded image which can be useful to confirm that the gridding and thresholding processes were accurate.

Upon satisfactory quantification of an image, a tab-delimited text file is saved which contains the area of each colony (in pixels) for each row and column position. The resulting data file can be viewed from within the program if so desired.

The colony measurement process can be performed in batch using a set of default parameters to process an entire folder of images. After doing this, a log file is generated which can be inspected to review any problems that occurred during analysis. On a typical test set up, a 1536 cpp plate scanned at 300 dpi takes approximately three seconds to analyze.

There are some additional features to aid with problematic images. If an image requires rotation, this can be achieved manually by a process which uses the positions of two colonies within the same row to determine the appropriate angle required to correct the orientation of the plate.

Any existing quantitation of a colony can be manually overridden by drawing an ellipse over the colony. The image can be zoomed to help with this. Additionally, if the particle finding algorithm rejected a particular grid location, it will be highlighted as a red square to draw attention to a position that may require this manual intervention. Colonies that have been manually defined are highlighted in magenta to differentiate them from those automatically quantified by the program.

### Data scoring: scoring tab

By default the scoring module will search the folder last used by the Image module to load files, although a different folder can be selected. The saved quantitation data files are analyzed to find sets of data corresponding to experimental plates. The user can then select which sets will comprise the control and experimental data and load them by selection from a drop-down menu. Upon loading, the software will normalize each plate of data and align the corresponding control and experimental data to produce paired sets.

The scored data can be saved in a variety of ways, listed either by colony grid position (recommended as it makes it easier to track specific colonies) or by the name of the ORF pertinent to each strain. In the case of multiple replicates of the same ORF, the mean area will be saved, along with the standard deviation. Additionally the user can select between two methods for saving experiments comprising multiple sets of plates. The recommended option is to save a separate file for each paired set of data as this retains the most information on individual colony sizes. However, it is also possible to combine multiple data sets, in which case the mean area and standard deviation will also be saved.

The scored data files also specify the gene at each position in the array. This requires a key file that maps the position of each colony in the array to a yeast ORF. The format of the key file is a tab delimited text file with four or five columns, with each row containing the following data:

•Column 1: plate number

•Column 2: row number

•Column 3: column number

•Column 4: systematic ORF name (e.g. “YLL040C”)

•Column 5 (optional): standard gene name (e.g. “VPS13”)

An example key file, “UBC-1536.key” is included with the program. If the gene name is not specified the software will attempt to determine this from the file “SGD_features.tab” which is found in the same folder as the program files. This file can be updated with the latest information from SGD from within the analysis module (see below). If the file is over 100 days old, the program will prompt to download a new version. The “Refresh Data” button will force the program to reload and score the selected control and experimental data files.

### Screen analysis

The final component of *Balony* is the Analysis module (Figure 2E). This enables the scored data from an experiment to be interrogated to identify positive and negative genetic interactions. The analysis module requires that each paired data set (control and experiment) is saved as a separate file as this ensures that quantitative data is saved for each individual colony. This is necessary for statistical analysis of colony sizes.

Users can elect to open all or a limited sub-set of scored data sets, which may be useful on occasions if there was a suspected technical problem with the plates of a particular set. After selecting files to load, the user is presented with a new window showing a table of the scored data. The data table will show averaged data for each array position along with the systematic ORF name and the standard gene name, the mean and standard deviation of the sizes of the control and experimental colonies at this position. The ratio (normalized experimental colony size/normalized control colony size) is displayed as an indication of the extent of any genetic interaction; this is followed by the number of replicates in which the ratio is either below or above a cut-off value.

The difference in colony sizes (normalized experimental colony size minus normalized control colony size) is also shown, which is analogous to the standard multiplicative score used in other protocols [7]. This is followed by the p-value obtained by performing a paired two-tailed t-test testing for a difference between the normalized colony sizes of the experimental strains vs. the normalized colony sizes of the control strains. Finally, whether this position is deemed to be a “hit” (see below) is indicated, followed by a column that will state if a position should be excluded from the analysis. These data can be sorted by any of these criteria.

In the case of SGA screens it is useful to define gene linkage at this stage and remove these genes from the analysis. This is accessed from the “Filtering” panel which opens a separate window displaying a graphical representation of the colony sizes of double mutants in genes that flank the query gene. Because of the reduced rate of recombination between linked genes (e.g. the query gene and an adjacent gene deletion), fewer double mutant cells are generated compared to unlinked genes, which leads to a characteristic decrease in colony size for these flanking linked genes [8]. The users then specifies the range of genes which are to be excluded from the analysis to prevent them from being reported as false positives. In addition, genes can be excluded manually by the user if they are known to be problematic or false positives for some other reason. Genes that have been excluded from the analysis can be shown or hidden using a toggle switch.

Analyses may benefit from discarding data where the control strain has very poor growth. In these cases it can be difficult to be sure a genuine synthetic lethal interaction is being observed when the growth of the control strain is particularly slow. This filtering can be based on the growth of the control strain, the experimental strain or both strains; this allows for flexibility if the read-out of a screen is something other than synthetic lethality. If the data is to be discarded, the size of both colonies for that paired set is set to zero to exclude them from further analysis.

In addition, maximal and minimal values may be assigned to colony sizes. This is useful if the ratio between colony sizes is being used as a measure of fitness. In the case of a strong aggravating (synthetic lethal) interaction, the double mutant may be essentially dead. However, there will still be a small amount of yeast present on the plate from the original pinning step. As this amount will vary between different colonies, this can lead to the impression that one synthetic lethal interaction is stronger than another, whereas in fact they are both reporting the same phenotype, i.e. no growth in the double mutant. By assigning a minimum value, all truly lethal interactions will have a similar score.

Following this, the user may wish to define cut-off values to define “hits”, which in the case of SGA experiments, are genetic interactions. While the difference between experimental and control colony size is often used as a measure for the strength of a genetic interaction, we have found that calculating the ratio of experimental to control colony size is a useful alternative. We find that this parameter is less influenced by the growth rate of control strains. For example, consider two strains *∆x* and *∆y* which grow with normalized colony sizes of 1.0 (i.e. as wild-type) and 0.4 respectively. If a second mutation is introduced so that the double mutant strain *∆x∆z* grows to a colony size of 0.8, and the *∆y∆z* strain grows to 0.2, it is clear that this mutation has had a greater effect on the *∆y* strain than *∆x* as it has led to a halving of the growth rate of the ∆y strain. However, if we were to report the difference between colony sizes, then both double mutant strains would yield a difference value of 0.2. In contrast, by using a ratio score, we find values of 0.8 and 0.5 for *∆x∆z* and *∆y∆z,* respectively, reflecting the relative strength of the observed genetic interactions.

Using the analysis function, two types of hits are distinguished between; those where the ratio is below a cut-off value (aggravating) and those where the ratio is above a second cut-off value (alleviating). If there is no genetic interaction, the ratio will be close to 1. A plot showing the distribution of ratios can be displayed to aid in screen validation. When first loaded, data files are sorted by array position, so that the distribution of ratios can be inspected to check for any systematic effects. Normally, it is expected that the variance between colonies would be distributed randomly, so if any trends are apparent, it is indicative of a systematic effect from either the pinning or imaging process. When arranged by ascending ratio this plot forms a characteristic curve with a steep initial portion representing aggravating interactions which levels off to a portion with a shallow gradient indicating no significant interactions, and then once again returns to a steep portion representing alleviating interactions. The software can estimate an appropriate cut-off value by extrapolating the linear central portion of the distribution and finding the *y*-intercepts at either end of the *x*-axis. Once these values have been determined, the data table will highlight “hits” in the screen based on criteria chosen by the user. The three criteria for a hit are:

1. The ratio is below the low cut-off value (*p*) or above the high cut-off value (*q*) as described above.

2. The number of replicates in which criterion (1) is met must be equal to or above a specified value (e.g. 3 replicates out of 3).

3. The p-value from a paired t-test of the sizes of the experimental colonies and the control colonies must be below a given value.

If all three criteria are met, then hits are highlighted in the table (green for those less than *p*, red for those greater than *q*). The table can be sorted to list these hits first, sorted by ratio from strongest to weakest.

From here, many users will find it useful to merely browse the list of genes. To aid in this, more detailed information can be readily accessed from the table. For each array position, a pop-up window can be displayed giving more detailed information on the corresponding. The normalized colony area will be shown for each individual colony, both numerically in a table; and as a graphical representation showing the currently highlighted control/experiment pair (as two solid circles) and the individual areas of all control and experimental colonies (as superimposed concentric circles). The description of the gene as defined by the SGD database is shown and this information can be kept up to date by downloading the latest database file from within the program. If the ratio plot is open, then the currently viewed query can be viewed on this plot to give an overview in the context of the entire screen. This window also contains a link to the corresponding page for the ORF in question on the SGD web site. The user can also quickly switch to positions containing duplicates of the current ORF elsewhere within the array, or to any other ORF of interest if present.

The context menu can be used from the table to select genes of interest to copy (either as ORFs, gene names, or both) for use in other applications or web sites. For example, a list of ORFs can be pasted into the gene ontology analysis utility at the SGD web site (http://www.yeastgenome.org/cgi-bin/GO/goTermFinder.pl). Additionally, the entire data table can be exported either as tab-delimited text, or as Microsoft Excel .xls file. This latter option prevents some formatting errors that can occur when importing tab-delimited text files into Excel, such as the interpretation of gene names as dates.

Users can also filter the list of genes displayed, based on a text string. Only those genes whose description contains this string will be displayed. This provides a quick way to check for interactions between genes involved in a particular function or process.

### Options

A fifth panel provides for setting of some basic options for operation of the program. This includes the ability to choose the type of user interface offered and a simple procedure to automatically update *Balony*.

## Results and discussion

### Colony size measurement

To speed up the quantification of plates with *Balony*, an automated gridding step can be used which attempts to automatically locate the position of the array, using a particle analysis routine to identify objects on the plate that resemble yeast colonies. To avoid counting extraneous plate features (e.g. bubbles, off-grid contaminants) as colonies, only a limited rectangular portion of the plate is scanned at one time. This region is based on the expected dimensions of the array from the grid preset. This routine generates a list of objects with defined *x* and *y* coordinates. The program assigns the objects closest to the corners of this region as the corners of the array, and then interrogates the spacing of all the other objects to see if they fit the criteria necessary to be colonies within the array. Specifically, if the *x* and *y* coordinates are both within 30% of an expected grid position, they are added to a list of valid colonies. After analyzing all the objects, the mean horizontal and vertical deviation of objects from their expected position is calculated and if each of these is within 5% of their expected values, then it is assumed that the array has been correctly established. If parameters are not determined from the initial analysis, the rectangle is progressively repositioned until parameters are correctly established. Occasionally, failure to automatically locate the grid may be due to a plate not being placed squarely in the scanner. The program contains an option to try a number of rotations to correct for this if the gridding process fails. This attempts to repeat the gridding process after rotating the plate by up to 3° in 0.5° increments. The current version of the algorithm was arrived at by repeated refinement using hundreds of test plates and we find it to be effective in for virtually all images we have encountered.

Once the grid has been successfully established, the sizes of colonies can be measured. This uses the same particle analyzer routine as used in the gridding. This generates a list of objects with *x* and *y* coordinates, areas and circularity values. The program iterates through this list, testing each object. A number of criteria must be successfully met for an object to be identified as a colony.

First, the centre of an object must be close to the centre of a grid position (by default both *x* and *y* coordinates must be within ¼ of a grid cell length, but this can be changed). Second, the colony must be within certain size limits, which again, can be specified. Finally, the colony must meet a minimum value for circularity, a parameter determined by the algorithm with possible values between 0 and 1, where 1 represents a perfectly circular object. The default minimum circularity value is 0.8. If more than one object is potentially allocated to the same grid position, the software will select the colony which is closest to the centre of the cell.

After interrogation of this list, the program individually analyzes the pixel content of any grid positions that did not have a colony allocated. If any position in the grid exceeds a minimum pixel count–suggesting the possibility of a colony that was not detected in the first pass–then the user is presented with the option to perform a low stringency second pass. In this case, the circularity of the particle finding algorithm is set to zero in order to identify “non-ideal” colonies; we find this helps to identify colonies that have, for example, become smeared during the pinning process.

**Figure 3:**
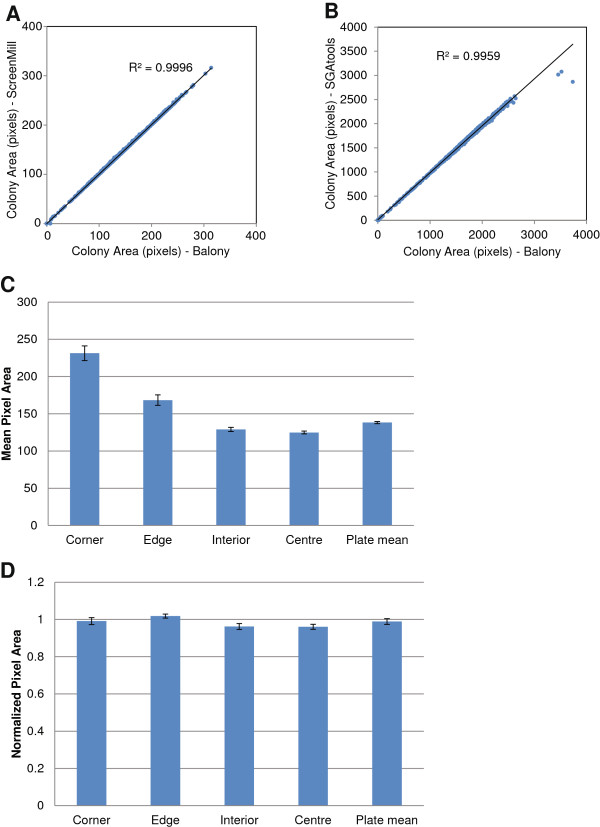
**Validation of colony measurement algorithm. A**. A plate image provided with ScreenMill was analyzed with Balony and ScreenMill and the measured size for each colony was plotted. **B**. As A, but comparing an image provided with SGATools. **C**. Eight YPD plates each containing 1536 colonies of wild type yeast (strain BY4741) were created and colony sizes measured by Balony. Colonies representing different positions on the plate were compared along with the mean colony size of each plate. Error bars show standard error. **D**. The same data as in C, but following normalization and “Row/Column” correction.

When we compared the ability of our software to measure colony sizes with ScreenMill and SGAtools, we found near-identical results. We analyzed a 1536-colony example plate provided with each program, and found the correlation between measured colony sizes gave a R[2] values of 0.9996 and 0.9959 respectively (Figure 3A, B), indicating that our implementation of colony measuring algorithms is similarly effective.

### Colony normalization

To demonstrate the fidelity of the normalization procedures employed by *Balony*, we constructed a test array plate of 1536 colonies of the same wild type yeast strain and scored colony sizes for eight replicates of this test array. First, we quantified growth of all 1536 wild type colonies across the eight plates, which should all have the same fitness. The mean pixel area per colony was determined to be 138.22 ± 1.52 pixels (Figure 3C). However, as previously described [9], we found that colonies at the corners and edges of the plate grew more quickly than colonies in the interior and at the centre (Figure 3C). This increased growth on the edges of the array is due to decreased competition with neighbouring colonies [9]. Thus, the non-normalized mean colony size measurement was not a reliable measure of colony fitness because it did not take into account systematic effects of array position on colony growth rate. In addition to the effects of array position, growth conditions can also vary substantially from plate to plate, further confounding attempts to quantify the fitness of individual colonies [9]. This was noticeable in our test array in which colonies in corner positions showed a wider range of areas, with a variation of up to 92 pixels across the eight replicates. In contrast, colonies in the centre of the array varied by only 17 pixels.

To circumvent these problems, we developed colony normalization algorithms which correct for decreased competition resulting from array position and for variability between plates. Colony normalization is an essential feature of all protocols developed for analysis of colony-based growth assays [9,10]. The first step in the normalization procedure employed by *Balony* is to divide the pixel area of each colony by the median colony size on each plate. Hence, a colony that grows near the average rate for that plate will have a normalized area of ~1. There are then three optional correction procedures that can be applied to the data, as described previously [9].

First, “Row/Column” correction can be applied. As we observed in our test array, the colonies at the edges of plates will often grow faster due to decreased competition with other colonies. To compensate for this, a correction factor can be applied based on the deviation of a given row or column compared to all other rows or columns. This is achieved by calculating the median pixel area of the spots in a particular row or column. If this value is greater than 1, then each spot in this row or column is divided by the median value to normalize that row or column with respect to the rest of the array.

The next type of correction is “Spatial” correction. This can be necessary in plates where the thickness of the media is variable because yeast colonies will grow at varying rates depending on the thickness of the media. To account for this, we take the median colony size of each row and column and fit these to a smoothed distribution using a LOESS algorithm [11]. This generates a pair of distributions, corresponding to the horizontal and vertical axes of the plate, with each value in the distribution expressed relative to the median colony size on the plate. From these distributions we can determine a correction factor for each position in the array, as the product of the corresponding row and column positions in each of the horizontal and vertical LOESS distributions.

The final type of correction employed by *Balony* is “Competition”. This can be necessary when a colony has a number of slow-growing colonies surrounding it. Due to this reduced competition for nutrients, that colony may then grow faster [9]. To control for this, we examine the whole plate for colonies whose eight surrounding neighbours have a mean growth rate of <75% of the median colony size. Using a simple linear regression analysis, we can determine if colony size correlates with the size of surrounding colonies on a given plate. If this correlation proves sufficiently robust (R^2^ > 0.1, slope between 0.1 and−1), then any colonies on the plate which have reduced competition (again, neighbours with a mean growth rate of <75% of the median) are corrected by applying the parameters derived from the linear regression to its colony size.

To confirm the effectiveness of colony normalization and Row/Column correction, we applied this algorithm to the raw pixel area data from the test array of wild type yeast shown in Figure 3C. While the raw pixel areas showed variations in colony size as high as 67% greater than the plate mean (for a corner colony), following Row/Column correction, each of the representative colonies reported a growth rate within 3% of the plate mean (Figure 3D). The low standard error associated with the mean corrected value for the plate (<5%) indicates that this algorithm effectively deals with variations caused by growth at the edges of plates. Thus, following normalization and Row/Column correction, we were able to determine with high accuracy that all colonies in the array had similar fitness, as would be expected since they are genetically identical.

**Figure 4:**
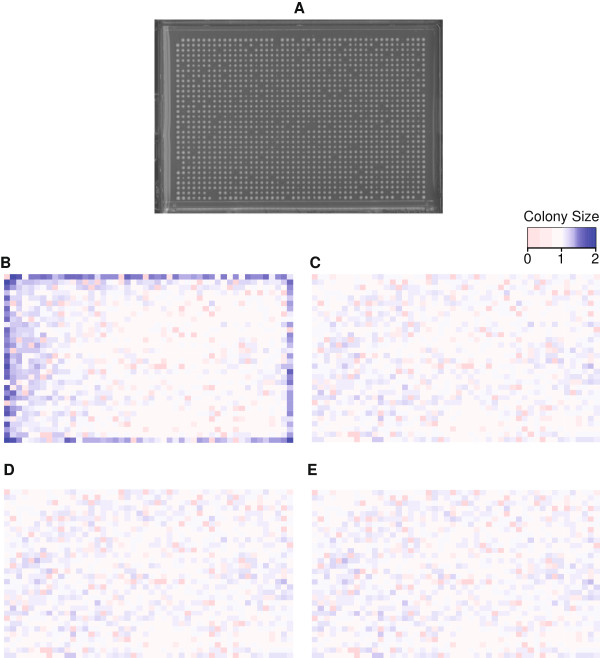
**Normalization of quantified colony measurements. A**. Scanned image of one of three replicates used in B-E. The shading of each cell represents the corrected colony size as indicated by the color key and each heatmap shows the mean of three biological replicates. **B**. Heatmap of uncorrected colony sizes. **C**. Heatmap after applying row/column correction. **D**. As C, but with spatial correction also applied. **E**. As D, but with competition correction also applied.

Now we compared the effects of each normalization algorithm using an actual 1536-density array plate of yeast single deletion mutants routinely used for SGA analysis in our lab. The effects of sequentially applying each type of correction are shown in Figure 4 (A-E). Applying Row/Column correction had the expected effect of normalizing the sizes of colonies in the outer rows and columns (compare Figure 4B & C). Subsequently applying Spatial and Competition correction resulted in much less dramatic corrections, likely because of the fairly uniform growth rates of the individual deletion mutants in the array (Figure 4C). For this reason we suggest that users only need to apply Row/Column correction unless they feel that their images would specifically benefit from the additional steps, such as in the case of unevenly poured plates, or with arrays containing a large number of slow-growing strains or empty spaces. As each correction step has the potential to distort the original data, we feel that it is beneficial to minimize the number of post-processing steps where possible.

### Genetic interactions of SCS2

To demonstrate the utility of this software, we performed an SGA experiment using a strain deleted for the gene *SCS2*. The analysis steps are described in a more detailed, step-by-step tutorial online at http://code.google.com/p/balony/wiki/Tutorial1 where a link to the scanned images is available should a user wish to follow the stages of analyzing a typical screen from start to finish.

**Figure 5:**
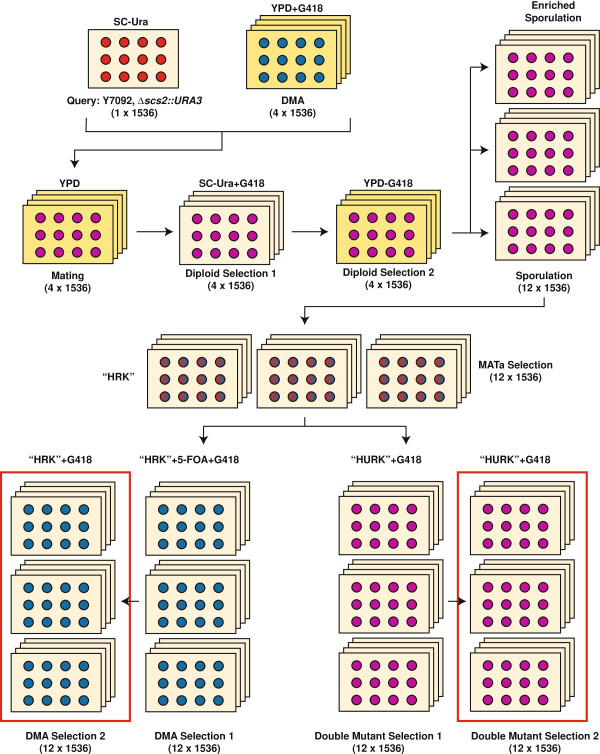
**Experimental procedure for an SGA screen for *SCS2*.** SC–synthetic complete medium; “HRK”–SC minus histidine/arginine/lysine, plus canavnine (100 mg/L) and thialysine (100 mg/L); “HURK”–as HRK, but minus uracil; 5-FOA–5-fluoroorotic acid.

The experimental approach is outlined in Figure 5. All robotic pinning steps were performed using a Singer RoToR HDA robot with colonies arrayed at a density of 1536 cpp. An *scs2::URA3* strain was constructed in the Y7092 strain background [2] and arrayed on SC-Ura plates. This was then mated with the DMA on YPD medium and diploids selected for on SC-Ura medium supplemented with 200 mg/L G418. After sporulation, *MAT****a*** haploid cells were germinated. We then generated a set of double mutants by two successive rounds of incubation on medium lacking uracil. Simultaneously, we generated single mutant control (DMA) strains by first incubating on medium containing 1 g/L 5-fluoroorotic acid (5-FOA) to counter-select for strains containing the *scs2::URA3* allele, and then incubating on medium containing uracil.

**Figure 6:**
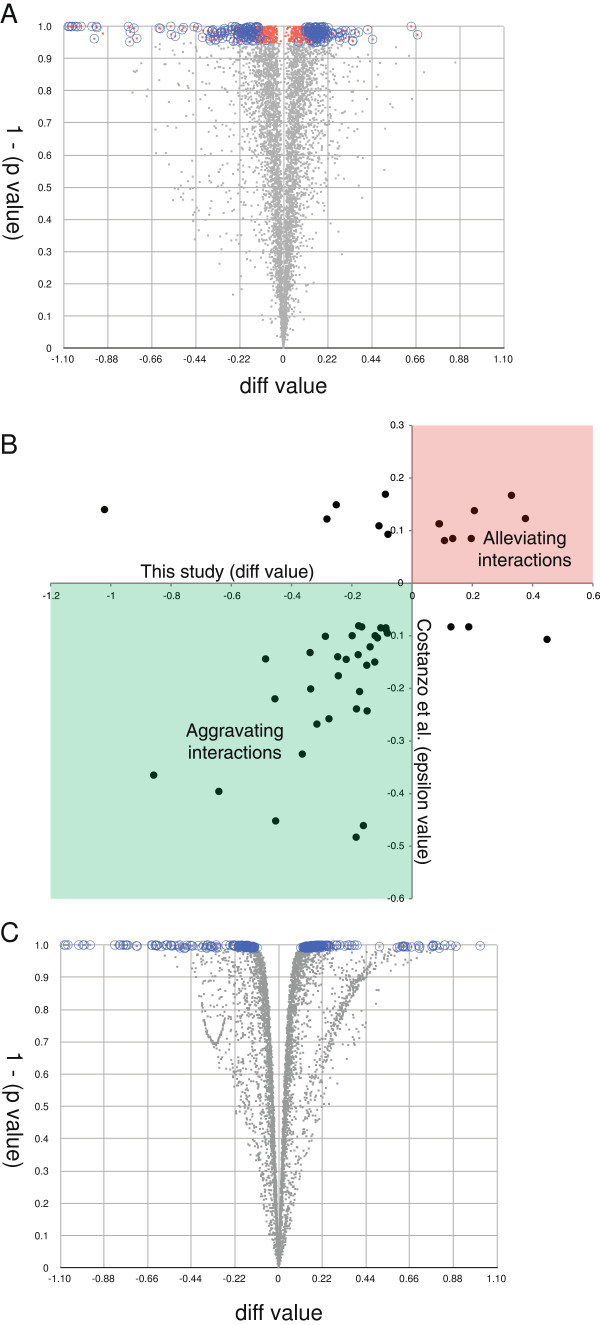
**Summary of our *SCS2 *SGA experiment. A**. Correlation of diff value with p value. Gray: p value > 0.05; red: p value < 0.05; circled red points: p < 0.05, diff above or below threshold in three replicates. **B**. Difference values in our screen plotted on the *x*-axis against epsilon values from the Boone lab data set on the *y*-axis. **C**. Correlation of diff value with p value using Bayesian analysis with Cyber-T. Gray: p value > 0.01; circled points: p value <0.01.

Each set of double and single mutant plates was scanned at 300 dpi and *Balony* was then used to analyze the images using the default image settings. Each paired set of data was scored using median Row/Column correction. We compared over 5500 single mutant control spots to the corresponding double mutant experimental spots in three biological replicates. We were able to identify significant differences in spot size for 638 experimental spots using a maximum p value of 0.05 (Figure 6A; red dots). Our ability to detect such a large number of potential interactions is a strong indication of the robustness of our methodology and of the high fidelity of the *Balony* colony scoring and normalization system. As expected, as the difference between control and experimental spot size approached zero it became increasingly difficult to define potential interactions with confidence (Figure 6A). As has been reported for previous analysis methods [10], we also identified a substantial number of experimental spots that exhibited only small differences in size from the corresponding control spot, but which were measured with unusually high accuracy (Figure 6A), which is likely an artifact of using only a small number of replicates (n = 3). However, these differences were unlikely to represent true genetic interactions because they fell within the 95% confidence interval of the mean difference measurements.

Using the analysis module of *Balony* to interrogate genetic interactions, we examined the ratios of spot sizes and used the ratio plot window to automatically define the ‘low’ and ‘high’ cut-off values for hits. The cut-off values obtained were 0.895 and 1.106, respectively. Using this method we were able to score 255 experimental spots that met these cut-off values in three out of three replicates that also had a maximum p value of 0.05 compared to the corresponding control spot size (Figure 6A; red dots within blue circles). This eliminated 383 experimental spots that showed only small, but significant differences from the corresponding control spot. Due to genetic linkage, a total of 68 spots corresponding to genes neighbouring *SCS2* were excluded from the analysis. Additionally, the *URA1*, *URA2*, *URA4*, *URA5* and *FUR4* genes were excluded as these mutants are involved in uracil metabolism and generate false “hits” due to the use of the–Ura selection media.

Using these ratios and a maximum p-value of 0.05 for the difference between control and experimental spot sizes, we identified 169 aggravating genetic interactions and 98 alleviating interactions in three out of three biological replicates. The list of genes responsible for these interactions was copied from the table and pasted into the FunSpec web site at http://funspec.med.utoronto.ca, to test for enrichment of this hit list for various gene ontology terms. We noted enrichment in a number of categories including “protein retention in Golgi apparatus” (p < 10^-4^), and “nuclear migration along microtubule” (p < 10^-4^) giving clues to the potential roles of the *SCS2* gene in the cell.

To determine how our analysis compared with previous SGA analyses we downloaded the data for the *SCS2* SGA screen performed by the Boone Lab [7] as part of their high throughput series of SGA experiments. To compare the data sets we used the “diff” value for the hits identified using *Balony* from our *SCS2* screen and the “epsilon” value from the Costanzo data set (Figure 6B). These are approximately equivalent measures of the strength of a genetic interaction as they compare the difference between the normalized colony size of a yeast double mutant and the corresponding single mutant control (see Conclusions for a more detailed definition). We found a high degree of overlap, with both aggravating and alleviating genetic interactions being found in both experiments. Of the 48 genetic interactions that were common to both screens (Figure 6B), 39 were found to have the identical effect, with 32 aggravating interactions and 7 alleviating. Using this information we were able to calculate values estimating the sensitivity and precision of our method, as follows:

The following formulae are used to define the parameters “precision” and “sensitivity” [7]:

$$\mathrm{precision}=\frac{\mathrm{TP}}{\mathrm{TP}+\mathrm{FP}}\;\mathrm{and}\;\mathrm{sensitivity}=\frac{\mathrm{TP}}{\mathrm{TP}+\mathrm{FN}}$$

where TP represents the number of “true positives” in the data set (genuine interactions correctly identified), and FP represents the number of “false positives” (interactions falsely identified).

Given that precision has been determined experimentally for the Boone lab data set at 0.63, and we know this paper reported 124 hits for this screen, which comprise a number of true positives and a number of false positives: Therefore, TP + FP = 124, so FP = 124-TP

$$\mathrm{precision}=\frac{\mathrm{TP}}{\mathrm{TP}+124-\mathrm{TP}}$$

So TP = 78 and FP = 46.

As the sensitivity of the Boone lab set has been estimated at 0.35, we can estimate the total number of genetic interactions for *SCS2* as 78/0.35 = 223. Our data set identified 32 genetic interactions in common with the Boone lab data set and these are likely to be genuine interactions; yet because the sensitivity of this data set is 0.35, this indicates that our data set contained a total of 32/0.35 = 91 true positive interactions. So of the 169 interactions identified, there are 169-91 = 78 false positives. The number of false negatives in our data set, i.e. interactions that we did not identify, must therefore be 223-91 = 132.

Applying the above formula, we were able to determine parameters for our screen which are summarized for comparison purposes in Table 2 alongside the values obtained from the Boone lab data set. The sensitivity of our screen compares well with that obtained by the Boone lab (0.41 vs. 0.35), as does the value we obtained for precision (0.53 vs. 0.63). This indicates that our protocol is sufficiently robust for routine laboratory usage. We speculate that these differences are largely due to two factors. First, the criteria used to distinguish hits are slightly different between the two methods, with our protocol relying on the ratio of colony sizes, with the Boone lab using the difference. Second, in our protocol we generate a control data set with each experimental data set, while the Boone lab uses a standard reference control set. These differences are likely to impact on the relative rates of false positive and false negative results obtained.

**Table 2:** Estimates of parameters for balony

	**Costanzo et al.**[7]	**Our data**
True positives	78	91
False positives	46	78
False negatives	144	131
Sensitivity	0.35	0.41
Precision	0.63	0.54

We also compared the *Balony* analysis method to an available method that uses a Bayesian framework for the analysis of biological data, which can be applied to any dataset that utilizes paired control and experimental measurements, and is particularly effective when there are only a limited number of replicates [12]. We used the Cyber-T program to analyze the colony size data for the *SCS2* SGA screen that was quantified and normalized using *Balony*, using the suggested parameters of an averaging window equal to 101, a Bayesian confidence value equal to 10, and a minimum p value of 0.01 [12].

We plotted the difference between control and experimental spot size versus the Bayesian p values and highlighted points with a p value below 0.01 (Figure 6C). As expected, this method increased the significance threshold for small difference measurements compared to t-test alone, eliminating spots with unusually low standard deviations due to having a small number of replicates. By this method 302 spots were identified that corresponded to 239 potential genetic interactions. We compared the genes identified by this method to the Boone lab data set and identified 14 genetic interactions in common, compared to 48 using *Balony*. Thus, making the assumption that the genetic interactions identified by the Boone lab were “gold standard” true genetic interactions, the algorithms employed by *Balony* appeared to be particularly well suited for the analysis of genetic interaction data derived from colony size measurements of high density yeast arrays.

## Conclusions

In this paper we have described a software package that makes the analysis of SGA data both rapid and flexible. We believe we have devised a complete system that can be employed at a relatively low cost, and in many cases will involve the purchase of no additional equipment. If necessary, the components for a dedicated imaging and computational platform (scanner, computer, high-resolution monitor) could be purchased for less than $2,000. It is our intention to continue development of the program in response to the needs of the community and to release regular updates offering new features.

Using the analysis features in *Balony* it is possible to determine parameters similar to those published for large scale data sets. Specifically, our diff measurement is analogous to the epsilon value. The epsilon value is defined as the difference between the observed growth of a double mutant strain and the predicted growth of the strain based on the relative fitness of each single mutant strain according to the multiplicative model for genetic interactions [13]. For example, in cases where the query strain has no associated fitness defect, then the difference measurement determined using *Balony* is equivalent to the epsilon value. However, should a query strain be used which does not grow as wild type, the difference measurement can be easily corrected to account for this. If a deletion strain is present in the DMA that corresponds to the query gene, then its growth can be used to approximate the growth rate of the query strain; otherwise the growth rate must be determined independently.

As a result it is possible for users of *Balony* to directly compare their results with the large resource of genetic interaction data already available. In this paper we have shown an example of this, comparing our *SCS2* SGA screen with the data available in public databases. The extensive correlations between the two data sets provide evidence that the analysis methods we have described here are sufficiently robust for routine analysis of genetic interaction data.

## Availabilty and requirements

**Project name:***Balony*

**Project home page:**http://code.google.com/p/balony/

**Operating system(s):** Platform-independent

**Programming language:** Java

**Other requirements:** Java 1.6 or higher, >1GB free memory.

**Licence:** GNU GPL

**Any restrictions to use by non-academics:** none

This site also hosts the source and a wiki which serves as a reference manual and contains a tutorial which guides a user through the analysis of a sample screen. This should also be consulted for details of system requirements and installation instructions.

## Competing interests

The authors’ declare that they have no competing interests.

## Authors’ contributions

BPY designed and developed *Balony* and wrote the paper. CJRL provided valuable input into the design of *Balony* and assisted with the program validation and writing the manuscript. Both authors read and approved the final version of the manuscript.
